# Monitoring depression with the PHQ-9 in primary care: a qualitative study

**DOI:** 10.3399/BJGPO.2025.0159

**Published:** 2026-03-25

**Authors:** Brian CF Ching, Rachel Dewar-Haggart, Carl R May, Geraldine Leydon, Tony Kendrick, Emilia Trapasso, Tasneem Patel, Molly Bird, Lauren Bridewell, Lien Bui, Emma Corcoran, Jane S Hahn, Riya Tiwari, Mekeda X Logan, Christopher Dowrick, Glyn Lewis, Mark Gabbay, Adam WA Geraghty

**Affiliations:** 1 Division of Psychiatry, University College London, London, UK; 2 School of Primary Care, Population Sciences & Medication Education, University of Southampton, Southampton, UK; 3 Faculty of Public Health and Policy, London School of Hygiene and Tropical Medicine, London, UK; 4 Department of Primary Care and Mental Health, University of Liverpool, Liverpool, UK

**Keywords:** depression, qualitative research, primary health care, general practice, patient reported outcome measures

## Abstract

**Background:**

Primary care guidelines recommend GPs consider using depression symptom questionnaires as patient-reported outcome measures (PROMs) to monitor depression in adults to inform treatment and evaluate management strategies. The PROMDEP randomised controlled trial assessed the use of the Patient Health Questionnaire’s (PHQ-9) effectiveness and cost-effectiveness for monitoring depression. We gathered qualitative evidence on the views and experiences of participating patients and practitioners to inform interpretation of the findings.

**Aim:**

To explore the views and experiences of patients and practitioners of using the PHQ-9 in the PROMDEP trial of monitoring depression in primary care.

**Design & setting:**

Nested qualitative study and process evaluation of the trial in primary care in England and Wales.

**Method:**

Twenty-nine patients and 15 practitioners took part in semi-structured telephone or video interviews. Interview data were analysed using thematic analysis.

**Results:**

Patients and practitioners both valued the PHQ-9 and reported limitations in its use for monitoring depression. This included its role in improving understanding of depression, impact on consultation and care, and integration within current primary care processes. In the context of the PROMDEP trial, our findings highlight potential reasons for the mixed trial findings, including how resistance in its use in practice may be owing to barriers that make it hard for practitioners to integrate the PHQ-9 in consultations.

**Conclusion:**

Monitoring of depression using PROMs needs to be considered within the context of current primary care processes and resources. Further research is warranted to understand how the PHQ-9 can be successfully integrated into consultations.

## How this fits in

Qualitative studies have shown mixed patient and practitioner views of using patient-reported outcome measures (PROMs) for depression. The PROMDEP trial was a cluster randomised controlled trial that found the Patient Health Questionnaire (PHQ-9) for the monitoring of depression did not incur significant benefit for depression scores in the short term. Our findings suggest potential benefits of using the PHQ-9 as a PROM for improving understanding of depression and impact on consultations and care, but also limitations in usability and implementation. The mixed trial findings of the PROMDEP trial may be explained by complex mechanisms underlying the use of the PHQ-9 in consultation.

## Introduction

Depression symptom questionnaires, such as the PHQ-9, have been recommended for use as PROMs in primary care depression guidelines internationally.^1–3^ However, despite its use across healthcare settings, there is mixed evidence about the effectiveness for the use of PROMs in monitoring depression in primary care.^4–7^ Following the National Institute for Health and Care Excellence (NICE) guidance in the UK between 2006 and 2013, the Quality and Outcomes Framework financially incentivised GPs to use symptom questionnaires to assess and follow-up depression. Qualitative research found that patients and GPs found PROMs acceptable to use and valued their utility in confirming diagnosis, facilitating structured conversations, and supporting decision making around appropriate treatments based on symptom severity.^5,8^ Conversely, some practitioners and patients felt PROMs intruded in consultations, hampered practitioner–patient dialogue, and were not motivated to implement their use in practice, and did not capture all depressive symptoms.^9–11^

Relatively few studies have investigated specifically the use of PHQ-9 for the monitoring of depression in primary care. The PROMDEP trial aimed to investigate the effectiveness of using PHQ-9 to assess and monitor depression in patients in UK practices.^12,13^ We found no significant benefit in depression scores at 12 weeks, suggesting that using PHQ-9 to monitor depression in practices were not significantly beneficial for depression in primary care patients. However, quality of life was better in the intervention arm at 26 weeks, and a clinically significant difference in depression scores at 26 weeks could not be ruled out. Importantly, only 41% of patients in the intervention arm had a GP follow-up PHQ-9 recorded. There may be unmeasured complex mechanisms not captured in the quantitative findings that may explain the mixed trial findings. To understand this, we also conducted a nested qualitative study and process evaluation within the PROMDEP trial to contextualise the quantitative trial findings.

In this article, we report the nested qualitative study and process evaluation, which aimed to explore the views and experiences of patients and practitioners of using the PHQ-9 to monitor depression in primary care, to inform greater understanding of the PROMDEP trial’s quantitative findings.

## Method

### The PROMDEP trial

The PROMDEP trial was a cluster randomised controlled trial (RCT) across 141 practices in England and Wales between November 2018 and December 2021. Practices were randomly allocated to the intervention or control group. Inclusion criteria were patients aged ≥18 years, with a new episode of depressive disorder or symptoms. Patients recruited into the study in the practices allocated to the intervention group had an initial PHQ-9 administered by a researcher within 2 weeks of recruitment. Practitioners, which included GPs or nurse practitioners, were then asked to repeat the PHQ-9 in a follow-up consultation in clinic. Patients were provided with an infographic with written feedback on their PHQ-9 scores and potential treatment options to discuss with their GPs after completing the PHQ-9 (see Supplementary Figure S1). Patients recruited into the control group received treatment as usual as provided by primary care practitioners. Further details of the trial and the PHQ-9 intervention are published elsewhere.^12,13^

The recruitment process and delivery of the PHQ-9 was impacted by the COVID-19 pandemic and changes were implemented during the trial as a result. Recruitment was significantly slowed down as practices were inundated and involvement in research activities were paused or not a priority for practitioners. As a result, recruitment took much longer than planned. Additionally, owing to national lockdown and social restrictions, the initial PHQ-9 was not administered in-person on paper by researchers and was instead completed digitally either over the telephone or via video conferencing. Primary care consultations also changed, with most practices offering remote consultations.

### Data collection

For the nested qualitative study and process evaluation embedded within the PROMDEP trial, we conducted interviews with participants. We recruited practitioners and patients in both trial arms using maximum variation sampling to capture perspectives from participants with a diverse range of characteristics, including gender, ethnicity, education level, and location of practice. Patients were interviewed between completing their 12-week and 26-week follow-up research assessments, and practitioners were invited during their participation in the trial. Patients gave consent to be contacted to take part in an interview as part of the consent process for the main part of the trial. Researchers approached potential participants by telephone and emailed the participant information sheet. Written, verbal, or electronic informed consent was obtained at the start of the interviews.

Interviews were completed by researchers (BCFC, RDH, ET, TP, MB, LBr, LBu, EC, JSH, RT, and MXL) and conducted face to face, by telephone, or online video call using Microsoft Teams. A semi-structured interview guide (see Supplementary Table S1) informed by normalisation process theory^14^ was used, developed by the research team and patient and public involvement (PPI) contributors. Interviews took place between August 2019 and July 2022. Interviews lasted 13–117 minutes. Interviews were audio-recorded and transcribed verbatim, with potentially identifying information removed from transcripts to ensure participant confidentiality and anonymity.

### Analysis

Analysis was conducted in two stages. First, we conducted thematic analysis.^15^ We sought immersion in the data by reading and re-reading all transcripts, reflecting on interviews, and discussing potential themes in data sessions. BCFC and RDH independently coded a set of transcripts and collaboratively developed an initial coding frame. This framework was then used to code subsequent transcripts, and was iteratively extended and revised as new codes were developed. Coding was inductive and derived from the data. BCFC developed the final set of themes from the coded data, refined with feedback from the research team and PPI representatives. Second, we undertook an attribution analysis^16^ of the results of the thematic analysis. CRM identified key attributions about the workability and integration of the intervention within the trial. These were then mapped onto the action constructs of normalisation process theory.^17^

We adhered to frameworks for conducting and writing up high-quality qualitative research.^18^ To maximise our result’s validity, multiple researchers were involved in the data collection, coding, and analysis. At different stages of the analysis, we presented preliminary findings to the research team and discussed the face validity of themes.

Our research team for this study is made up of a diverse group of academic and clinical researchers at different career stages, including research assistants, associates, and professors. Study team members have expertise across multiple clinical specialties, including general practice and psychiatry, and have contributed to the development of the NICE guidelines for the treatment and management of depression in adults. We bring a range of research interests and experiences into the study, including qualitative methods, development and evaluation of complex interventions (including PROMs), implementation science, and primary care service development. We represent a range of gender, culture, and racial identities (including Black, Asian, and White British). This range of identities and perspectives enhanced our knowledge and insight on experiences of depression and its monitoring in primary care, but we still maintained curiosity and reflected on our preconceived notions, allowing diverse ideas to be developed inductively from the data. These important reflections informed how we interpreted and analysed the qualitative data.

## Results

In total, 29 patients and 15 practitioners agreed to be interviewed for the study. Tables 1 and 2 show participant characteristics for patients and practitioners. Five inductive themes were developed after analysis of all interview data, which are discussed below (see Table 3). We included quotes to illustrate and evidence our analysis. Quotes have been allocated participant identification codes to maintain confidentiality and anonymity (GP for practitioners and PT for patients).

**Table 1.** Participant characteristics: patient interviews (*N* = 29)

Characteristic	Intervention, *n* = 18, *n* ^a^	Control, *n* = 11, *n* ^a^
**Gender**		
Woman	10	8
Man	8	3
**Age, years, mean (SD)**	36.7 (12.7)	46.8 (19.7)
**Ethnicity**		
White	15	10
Chinese	1	0
Indian	1	0
African and Irish	1	0
Persian	0	1
**Marital status**		
Married or cohabiting	7	7
Single	11	3
Divorced	0	1
**Education level**		
None	2	0
CSE or NVQ Level 1	1	0
GCSE, O Level, or NVQ Level 2	3	1
HNC, HND, City & Guilds, Teaching qualification, or NVQ Level 4	2	1
Degree, higher degree, or NVQ Level 5	10	7
Vocational qualification	0	2
**Employment status**		
Full-time work or self-employed	11	5
Part-time work	2	5
Homemaker	1	0
Retired	1	1
Student	3	0
**IMD, median**	7	9

^a^Unless otherwise specified. CSE = Certificate of Secondary Education. GCSE = General Certificate of Secondary Education. HNC = Higher National Certificate. HND = Higher National Diploma. IMD = Index of Multiple Deprivation. NVQ = National Vocational Qualification. SD = standard deviation.

**Table 2.** Participant characteristics: practitioner interviews (*N* = 15)

Characteristic	Intervention, *n* = 11, *n* ^a^	Control, *n* = 4, *n* ^a^
**Gender**		
Woman	4	3
Man	7	1
**Location**		
Urban	8	3
Rural	3	1
**Practice size**		
Large	7	3
Small	4	1
**IMD, median**	7	8

^a^Unless otherwise specified. IMD = Index of Multiple Deprivation.

**Table 3.** Themes and sub-themes

**Theme**	**Sub-theme**
1. Improved understanding of depression	1.1 Recognising symptoms
	1.2 Monitoring over time
	1.3 Motivation and hope
2. Usability	2.1 Being pigeonholed
	2.2 Accessibility
3. Impact on the consultation	3.1 Driver of discussion
	3.2 Patient–GP relationship
	3.3 Person-centred care
4. Impact on care	4.1 Evidence to inform treatment and management
	4.2 Objectivity versus subjectivity
5. Organisational barriers and facilitators to implementation	5.1 Time restraints
	5.2 Technological integration
	5.3 Frameworks and guidelines

### Thematic analysis

#### Improved understanding of depression

##### Recognising symptoms

Practitioners and patients believed the PHQ-9 was a helpful tool in providing information on the range of symptoms and severity categories of depression and how particular symptoms affected them:

*‘Having your depressive symptoms split up into nine sections I think is helpful. It’s not a big accumulation of just feeling horrible, it’s splitting it up and making sense of it. I think it works for lots of people who want to understand their symptoms better.’* (GP01002-01)

*‘As I was reading* [the items] *I was thinking to myself — yes, that is me, that is me and I kind of understood* [depression] *bit more.’* (PT02024-03)

Some patients felt that seeing each item and the infographic was validating, which highlighted that they needed support, and provided solace that they were not alone:

*‘It reassured me that I wasn’t going mad, that it is an illness, and it does affect a lot of other people as well.’* (PT01023-02)

##### Monitoring over time

The PHQ-9 was reported to be a useful tool for practitioners to monitor their patients’ depressive symptoms temporally, identifying improvements and deteriorations. Regularly completing the PHQ-9 highlighted changes in depressive symptoms, which was reassuring and rewarding to patients, allowing patients to ‘map out’ areas for improvement:

*‘It’s mainly mapping to see which bits needed to be improved and which bits could be not focused on for a little while, what to work on or how to make yourself feel better.’* (PT03037-03)

In the control group, patients described how they were monitored over time without questionnaires. They described how practitioners used the conversation in the consultation to check in, using both what the participants said and how they appeared to look for worsening or improvement:

**PT:** ‘*She said I looked like I was improving, like mood - wise.’*

**Interviewer:** *‘Yes, it sounds like she’s kind of gauged that from the mood and in the consultation perhaps?’*

**PT:** *‘I think a combination of both, really, yes, I guess maybe how I was like saying these things on top of what I was saying.’* (PT 0305103)

##### Motivation and hope

Using the PHQ-9 and realising the severity of their depression motivated some patients in actively seeking support. Over time, seeing improvements motivated patients to continue their efforts and emphasised that their perseverance was worthwhile, providing hope:

*‘When I see it go down, you're relieved. You're like, oh, it’s working; it’s worth getting help.’* (PT01002-04)

### Usability

#### Being pigeonholed

Some practitioners and patients experienced the PHQ-9 as limited in capturing people’s experiences and symptoms, suggesting response options on the PHQ-9 pigeonholed patients into arbitrary categories. This was perceived by patients as inaccurate and reductive of their complex experiences:

*‘The frequency of symptoms remained the same, but the intensity decreased. I felt like I couldn’t reflect it on the questionnaire.’* (PT01096-05)

#### Accessibility

Some practitioners suggested, that beyond the trial, the waiting room was a prime location to complete the PHQ-9. Staff in reception could support those who need help filling them in and this would ensure patients complete the PHQ-9 as asked. Especially for patients who *‘can’t motivate themselves to get out of bed, they may not get the motivation to do the form’* (GP01026-01).

### Impact on the consultation

#### Driver of discussion

As each item highlighted a specific symptom, practitioners felt that the PHQ-9 was helpful in identifying specific symptoms patients were experiencing and their impact. The PHQ-9 reminded them to conduct necessary risk assessments. This enabled safety netting and necessary referral to mental health services, and prompted practitioners to have sensitive conversations with patients:

*‘You are forced to ask the question about the self-harm ... In a consultation where you have other things to discuss with them, it can be easy to forget.’* (GP01002-01)

The PHQ-9 was described as being helpful in guiding discussions around patients’ difficulties, needs, and care. It facilitated productive conversations as all parties had information regarding the problem as assessed by the PHQ-9:

*‘It sometimes is really difficult to explain how you're feeling. Being able to put it down on paper like that both yourself and your GP can see how you have been feeling and what areas are a bit worse than others.’* (PT01002-04)

For some patients, the PHQ-9 facilitated conversations about depression, which was difficult to speak about owing to reasons such as stigma, feeling like they could not articulate their experiences, or the perception that the GP practice was not a place for mental health.

#### Patient–GP relationship

The PHQ-9 facilitated practitioners’ engagement with and support to patients through validation, praise, monitoring, and maintenance of progress:

*‘If the PHQ-9 score was decreasing, then I would congratulate the patient … I say, that’s great progress.’* (GP02013-01)

The structure provided by the PHQ-9 ensured patients could develop a trusting and personal relationship with their GP and be reassured they were supported. Continuity of care was voiced by patients as paramount here, as they could build rapport with their doctor and not need to repeatedly retell their problems:

*‘It was good knowing that there’s somebody there to monitor that progress. I think that’s reassuring.’* (PT03044-04)

#### Person-centred care

Practitioners described how the PHQ-9 provided a richer understanding of how depression impacted their patients. The PHQ-9 also facilitated conversations between practitioner and patient about their care based on what patients want.

*‘I think it’s a more holistic assessment than you would do if you're left to your own devices and you ask just three random questions, which might not actually show you the whole extent of how things affect you ... I negotiate with the patients what they think might be acceptable to them.’* (GP01002-01)

### Impact on care

#### Evidence to inform treatment and management

Most practitioners and patients found the PHQ-9 solidified decisions about potential treatments and informed the urgency of suggestions, where worse scores would indicate more intensive treatment and insistence:

*‘I perhaps might be more persuasive towards someone who’s dark red saying I think we do need to start you on treatment … If someone’s yellow, I might sit more on the fence and just say — this is an option, how do you feel? Would you prefer to have treatment or not have treatment?’* (GP01056-01)

The PHQ-9 score was also indicative of whether the treatment plan was working. If scores did not change or increase, this made practitioners and patients consider stepping up treatment.

#### Objectivity versus subjectivity

Many practitioners saw the PHQ-9 as an objective measure in providing clarifying quantitative information about patients’ difficulties and needs where symptoms may not present the same or as clearly in all patients. The PHQ-9 was deemed useful for patients where symptoms may not be overtly visible during consultation:

*‘*[The PHQ-9] *is something we should think about using, especially with something as grey as depression … People could be tearful but not have really bad depression and there could be other people who look quite together with it but actually who were severely depressed. It’s a helpful, objective tool.’* (GP03044-01)

Other practitioners, especially those with more experience, preferred using their clinical judgement in making decisions about patients’ care. They did not like the rigidity of the categories and their suggested treatments as it felt like *‘tick-box medicine’* (GP01002-01). These practitioners expressed resistance to the continued use of the PHQ-9. However, they suggested it could be used as a guidance for younger practitioners with less experience:

*‘Doctors as a rule don't like tick-box medicine unless they are starting out as new doctors and haven't found their feet yet ... As you're becoming more senior, doctors tend to do less of those things, because they feel they are being patronised and being told what to do ... They'd rather do what they think is right rather than somebody else telling them to do so.’* (GP01002-01)

### Organisational barriers and facilitators to implementation

#### Time restraints

Time limitations for each consultation was identified as a huge barrier to practitioners’ use of the PHQ-9. Within a 10-minute consultation, GPs felt unable to spend time administering or discussing the PHQ-9 on top of general discussions around the patients’ depression:

*‘They'll be given a ten-minute appointment which is the usual in GP land … Which is a bit of a shame when assessing depression because it clearly takes longer than that, let alone then having to do a PHQ-9.’* (GP01026-01)

#### Technological integration

Beyond the trial processes, for practitioners where the PHQ-9 was embedded within their practices’ digital systems, benefits were perceived, including increased access to the PHQ-9, efficiency of appointment distributions by need, and maintained privacy for patients. This may have preserved resources and promoted better care:

*‘We can just text them the PHQ-9 link and they can fill it out. We can ask them to do the score once a month and they can text it or email it to the surgery. It gets automatically downloaded on the patient notes.’* (GP02013-01)

#### Frameworks and guidelines

Many practitioners felt that if the PHQ-9 was to be recommended in national guidelines they would feel more encouraged to implement it in practice. To facilitate this, practitioners would need access to a clear evidence-base for its effectiveness for depression monitoring:

*‘If it was part of NICE guidelines, that would be different. We have to adhere to them … I think you find most people won't have a better system to assess patients, certainly not an evidence-based system. That probably would be the single best way to get it into the general GP population and use it in practice.’* (GP01002-01)

### Attribution analysis

Thematic analysis provided a robust account of factors that promote or inhibit incorporation of the PHQ-9 in clinical practice. We then moved to an attribution analysis (see Table 4 for key attributions mapped onto action constructions of normalisation process theory). In the PROMDEP trial, the PHQ-9 conferred interactional advantages on patients because it provided them with a tool that enabled them to make sense of their depression, even though it captured only a limited range of their experienced symptoms. It also conferred an advantage on inexperienced practitioners because it provided an objective measure of symptom severity. However, within the trial, there was evidence that the PHQ-9 was time-consuming and difficult to integrate into the consultation. Practitioners’ accounts suggested that it could be pushed out of the in-person consultation and configured as an element of e-consult or could be administered by receptionists or nurses.

**Table 4.** Attribution analysis mapped onto action constructions of normalisation process theory^a^

NPT action constructs	Usability of the PHQ-9	Impact on the consultation	Improved understanding of depression	Impact on care	Other factors that promote or inhibit implementation	Conclusion
*Interactional workability:* **how does the PHQ-9 affect interactions between patients and care processes?**	(+) assists patient in (subjective) recognition of symptoms	(+) validates patient’s need for care	(+) allows patients to understand severity of symptoms and actively seek support	(+) allows patients to ‘map out’ areas for improvement	(-) assumes that patients are motivated to effectively participate in their care	**PHQ-9 has an interactional workability advantage for patients** ++++ Normalisation potential
*Relational integration:* **how does the PHQ-9 relate to existing knowledge and relationships?**	(+) supports inexperienced practitioners in (objective) diagnostic process	(+) assures patient of continuity of support from practitioners	(-) results may be perceived by patients as inaccurate and reductionist	(-) captures limited range of patient experiences	(+) allows practitioners to effectively assess urgency of presentation	**PHQ-9 has a relational integration advantage for less experienced practitioners, but not for patients or more experienced practitioners** +++ Normalisation potential
*Skill-set workability:* **how is the character of patient and practitioners work affected by the PHQ-9?**	(+) provides a sense-making tool for patients and practitioners	(+) can shift patient work to the waiting room, e-consult, or online	(+) supports practitioners’ clinical judgement	(+) permits monitoring of changing symptoms over time	(+) allows risk assessments around self-harm and permits objective referrals to mental health services	**PHQ-9 has a skill-set workability advantage for practitioners** +++++ Normalisation potential
*Contextual integration:* **how does the PHQ-9 relate to the setting in which it is operationalised?**	(+) changes in scores show whether practitioners’ treatment plan is working	(-) adds time and complexity to the consultation	(+) score links to evidence-based treatment suggestions	(-) needs to be embedded in digital systems, adds to efficiency of access	(-) not included in national guidelines	**PHQ-9,** *in the trial* **, has clinical value but is poorly contextually integrated in practice** ++ Normalisation potential

^a^(+) and (-) indicates positive mechanisms or facilitators and barriers to implementation of the PHQ-9 in the monitoring of depression in primary care. NPT = normalisation process theory. PHQ-9 = Patient Health Questionnaire.

## Discussion

### Summary

Our nested qualitative study and process evaluation highlighted that patients and practitioners reported various benefits of using the PHQ-9 to monitor depression in primary care. Particularly, the PHQ-9 provided information on range and severity of depression symptoms, temporal changes in mood, informed treatment plans, facilitated dialogue, ensured risk assessments were carried out, and promoted a trusting practitioner–patient relationship and person-centred care.

However, the PHQ-9 was also perceived to have limitations. Some patients expressed the PHQ-9 as limited in capturing their nuanced experiences and meaningful changes. Practitioners also described the PHQ-9’s severity categories and treatment suggestions as *‘tick-box medicine’*. There was also resistance in some practitioners to its use in practice.

In the context of the PROMDEP trial, these findings shed light on potential mechanisms that may explain the quantitative findings. The trial reported no significant effect on depression scores at 12 weeks. Use of the PHQ-9 in clinics may not have translated to changes that positively impacted depression outcomes owing to factors related to usability and organisation. Practitioners highlighted issues with the integration of the PHQ-9 into current primary care systems and consultations. This is relevant considering a proportion of patients in the intervention arm did not have an initial or a follow-up PHQ-9 recorded, suggesting some patients in the intervention arm were not provided with the PHQ-9 itself. Inconsistent fidelity across GPs who were provided with compensation and training to administer the PHQ-9 suggests that the use of PHQ-9 as a depression monitoring tool involves a myriad of complex mechanisms that need to be addressed in primary care. This includes practitioner perceptions of the PHQ-9, guidelines and frameworks, and how it fits with current processes and technology in practices.

### Strengths and limitations

Our nested qualitative study and process evaluation purposefully sought a diverse group of patients and practitioners. We explored a wide range of patient and practitioner experiences and views, which maximise the potential transferability of our findings. We also harnessed the diversity of our study team in the data collection and analysis, which provided unique perspectives and input from different backgrounds (for example, gender and racial identities) and expertise (for example, clinical and academic roles). Rigorous qualitative methodology was followed to ensure validity and trustworthiness of our findings. Our study provided in-depth detail of potential mechanisms that may underly the findings of the main PROMDEP trial. We demonstrate that the use of PHQ-9 in monitoring depression in primary care is a complex intervention that requires integration into current processes for successful uptake by practitioners.

Potential biases may have been present in our recruitment. Patients and practitioners with more positive opinions about the PHQ-9 may have volunteered to participate as only interested participants were recruited into this study. Alternative experiences and views about the PHQ-9 may have been left out. Additionally, despite sampling efforts, certain characteristics dominated our sample (that is, White and employed). Experiences of the PHQ-9 and monitoring of depression in primary care may vary across minoritised identities,^19^ which refers to groups that have intersecting identities that may be socially minoritised and disadvantaged, such as women, people of colour, and people in deprived communities.^20^ Other characteristics that may have been important in the experiences of using the PHQ-9, such as practitioner job role, were not collected. This needs to be considered in interpretation of our findings.

### Comparison with existing literature

Our findings complement previous studies that suggest potential utility of using PROMs in monitoring depression in primary care. Previous research found that PROMs helped patients identify and understand their depressive symptoms, provided structure to the consultation, and supported practitioners in developing and confirming treatment decisions, and monitoring changes in depressive symptoms.^5,8^ Our study extends this by evidencing its utility in also instilling hope of recovery in patients, and supporting patients and practitioners in monitoring changes in depressive symptoms, developing a trusting and communicative relationship, and fostering person-centred care. However, this needs to be interpreted within the context of low fidelity within the PROMDEP trial, suggesting that this benefit may only be translated in practices when practitioners feel able to integrate this within their consultations. When implementing new methods or components of primary care consultations, adding to clinical workloads of practitioners is a barrier in its use.^21^ Malpass *et al* found that some patients experienced the PHQ-9 as limited in accurately capturing their depressive symptoms owing to the limited scope of the items.^10^ Additionally, research suggests that practitioners prefer using their clinical judgement (‘phronesis’) over PROMs in consultations as they value the human element of the practitioner–patient dialogue.^7^ Our study additionally highlighted how those views may vary depending on years of experience, where older practitioners may perceive the need to use the PHQ-9 as patronising and a burden. Our findings suggest that patients want to be able to identify the impact or magnitude of symptoms, which the PHQ-9 may be limited in monitoring, and practitioners need to be inherently motivated to use a tool for its regular use.

### Implications for research and practice

The quantitative findings of the RCT were that the intervention arm practitioners recorded follow-up PHQ-9 results in the medical records of only 41% of patients, suggesting that questionnaires were not used routinely to inform treatment decisions by all practitioners, even in practices that had volunteered to use them in the trial.^12,13^ It is possible the increased demands on practices during the COVID-19 pandemic made the completion of the study a lower priority during 2020–2021.

Using the questionnaire could be made easier than it was in the trial. The extra time taken in consultations could be considerably reduced by automatic remote administration of the questionnaire beforehand, through sending it by text message or email, or including its administration in online consultations (e-consults). Some practice computer systems already incorporate a PHQ-9 automatically in e-consult questions for patients who indicate they may have mental health problems. Alternatively, telephone administration of the PHQ-9 has been shown to be reliable,^22^ and could be done by other practitioners or even administrative staff whose time is less expensive than that of GPs.

Future research is warranted to better understand how uptake of PHQ-9 and related PROMs can be improved in primary care. Rigorous and thoughtful implementation research needs to be conducted and consistently incorporate patient and practitioner perspectives, as well as other important stakeholders, such as service planners and policymakers. Considerations need to be made within current primary care systems and infrastructure.

Monitoring of depression using PROMs needs to be considered further within the context of the current drive towards digitalising primary care processes and increasingly stretched resources. Successful integration of the PHQ-9 needs to be facilitated in ways that increase practitioners’ decision-making capacity and promote positive perceptions about PROMs, including enablement of the seamless use of PROMs in time-limited consultations.

Crucially, our quantitative and qualitative evidence shows questionnaire monitoring is useful for increasing patients’ understanding of the wide range of depressive symptoms, and that patients like to track their own symptom changes over time. Practitioners should recognise those advantages, even if the scores do not directly inform their treatment decisions.

## Trial registration number

Trial registration: ISRCTN 17299295.

## Data

Our research ethics permission is for use of the data by the research team only. Applications to use the data need to be in the form of a peer-reviewed protocol, and will be considered by the three lead investigators, Tony Kendrick, Glyn Lewis, and Mark Gabbay, in the first instance, before a decision on data sharing will be taken by the rest of the co-authors. Qualitative data will not be shared, due to the relatively high risk of breaches of confidentiality arising from the nature of qualitative interviews, which are difficult to make completely anonymous.
